# Frailty and Processing Speed Performance at the Cusp of Midlife in CATSLife

**DOI:** 10.1093/geroni/igab046.2624

**Published:** 2021-12-17

**Authors:** Maria Luna, Shandell Pahlen, Robin Corley, Sally Wadsworth, Chandra Reynolds

**Affiliations:** 1 University of California, Riverside, Riverside, California, United States; 2 University of California, University of California, California, United States; 3 University of Colorado Boulder, Boulder, Colorado, United States; 4 University of California Riverside, Riverside, California, United States

## Abstract

Frailty is an important multi-domain measure of health status and aging. Processing speed (PS) performance may be predictive of later frailty among older adults, but the interrelation between frailty and PS at the cusp of mid-adulthood is unclear. Using data from the ongoing Colorado Adoption/Twin Study of Lifespan Behavioral Development and Cognitive Aging (CATSLife; N = 1213; Mean age = 33.22 years; SD = 5.0), we constructed a 24-item frailty sum score across anthropomorphic, objective health, and perceived health and engagement measures based on the Accumulation of Deficits model. PS was measured using the Colorado Perceptual Speed (CPS) and WAIS-III Digit Symbol (DS) tests. All mixed-effects regression models accounted for clustering among siblings, and covariates included sex, age, race, ethnicity, and educational attainment. Intraclass correlations (ICCs) [95% CI] for frailty among siblings, adjusted for sex and age, ranged from near zero for siblings in adoptive families, .13 [.08-.30] for nonadoptive siblings/fraternal (DZ) twins, and .44 [.40-.48] for identical (MZ) twins, suggesting possible heritable influences. Poorer PS performance was associated with higher frailty, but was significant for DS only (B: DS = -0.43, p =.005). Furthermore, the DS-frailty association was magnified by age (B: DSxAge = -0.06, p =.025), suggesting that the associations between processing speed and frailty may increase with age. These findings help elucidate the interrelationship between indicators of frailty and cognitive performance for adults approaching midlife, a salient and understudied period within lifespan development.

